# Strengthened Optical Nonlinearity of V_2_C Hybrids Inlaid with Silver Nanoparticles

**DOI:** 10.3390/nano12101647

**Published:** 2022-05-12

**Authors:** Yabin Shao, Qing He, Lingling Xiang, Zibin Xu, Xiaoou Cai, Chen Chen

**Affiliations:** 1 School of Jia Yang, Zhejiang Shuren University, Shaoxing 312028, China; shao_yabin@163.com (Y.S.); melody.xiang@vip.163.com (L.X.); xu_zibin@163.com (Z.X.); cai.xo@163.com (X.C.); 2 Collaborative Innovation Center of Steel Technology, University of Science and Technology Beijing, Beijing 100083, China; 123simon@163.com; 3 College of Civil Engineering, East University of Heilongjiang, Harbin 150086, China

**Keywords:** Ag@V_2_C MXene, hybrids, Z-scan, nonlinear optical properties

## Abstract

The investigation of nonlinear optical characteristics resulting from the light–matter interactions of two-dimensional (2D) nano materials has contributed to the extensive use of photonics. In this study, we synthesize a 2D MXene (V_2_C) monolayer nanosheet by the selective etching of Al from V_2_AlC at room temperature and use the nanosecond Z-scan technique with 532 nm to determine the nonlinear optical characters of the Ag@V_2_C hybrid. The z-scan experiment reveals that Ag@V_2_C hybrids usually exhibits saturable absorption owing to the bleaching of the ground state plasma, and the switch from saturable absorption to reverse saturable absorption takes place. The findings demonstrate that Ag@V_2_C has optical nonlinear characters. The quantitative data of the nonlinear absorption of Ag@V_2_C varies with the wavelength and the reverse saturable absorption results from the two-photon absorption, which proves that Ag@V_2_C hybrids have great potential for future ultrathin optoelectronic devices.

## 1. Introduction

MXenes [1], 2D transition metal carbides and nitrides, exhibit the outstanding advantages of electrical conductivity, a beneficial elastic modulus and capacitance, an adaptable band gap and optical transparency [2,3,4,5,6]. Nonlinear optical (NLO) properties of MXene have drawn wide attention [7]. Among them, Ti_3_C_2_Tx is the first and most-studied MXene, and the research has been progressing vigorously regarding the linear optical properties [8,9,10,11,12,13].

Ti_3_C_2_Tx thin films was studied by Mochalin and Podila et al., and the findings exhibited a high modulation depth of 50% and high damage thresholds of 70 mJ cm^−2^ [11]. Ti_3_C_2_Tx was synthesized by Wen and Zhang et al. who revealed the Ti_3_C_2_Tx maximum nonlinear absorption coefficient 10–21 m^2^/V^2^ had been increased tremendously compared with other 2D materials [12]. 

Thus far, the linear optical properties have been explored experimentally and theoretically in an extensive way, while little attention has been drawn to V_2_CT_X_‘s optical nonlinear features as well as its related applications. The Z-scan technique proposed by Sheik-Bahae et al. is simple and accurate and is widely used to study the nonlinear properties of materials (particularly non-fluorescent) [13]. 

To achieve the application-oriented demands for MXenes, a large variety of modified techniques have been invented to obtain desired functionalities, such as the previous studies on colloidal solutions of nanoparticles (NPs) hybridized by graphene, TMDs, etc. [14].

Moreover, great breakthroughs have taken place in NLO applications via hybridizing NPs in the past few years in accordance with their defect states, surface control and excellent plasmonic properties [15,16,17]. In various experiments on its broadband and strong NLO response, V_2_CT_X_ nanosheets, a new member of the MXene family, has drawn extensive attention due to its novel optical properties. Recent studies have shown that it has very good performance in hybrid mode-locking and good optical nonlinear properties, which indicates its good potential applications in acting as sensors and nonlinear components for lasers [18,19].

## 2. Materials and Methods

### 2.1. Preparation of Ag@V_2_C Hybrids

First, 400 mesh V_2_AlC (1 g) powder (Suzhou Beike Nano Technology Co., Ltd., Suzhou, China) is added slowly to 50% (*v*/*v*) HF solution, and it is stirred at room temperature for 90 h [16,17,18] to ensure the V-Al bonds are completely disconnected from V_2_AlC with the V-C bonds intact. Second, to stratify the V_2_CT_X_, 30 mL tetraethylammonium hydroxide (TMAOH 25% in H_2_O) is blended with 1 g multilayer V_2_CT_X_ and sonicated for 30 min at room temperature, and the precipitates are rinsed with deionized water to neutrality (pH ≥ 6). Then, the excess TMAOH is discarded from the product by repeated centrifugation at 3000 rpm. Finally, a 2D monolayer V_2_CT_X_ MXene material is obtained via freeze-drying.

**Figure 1:**
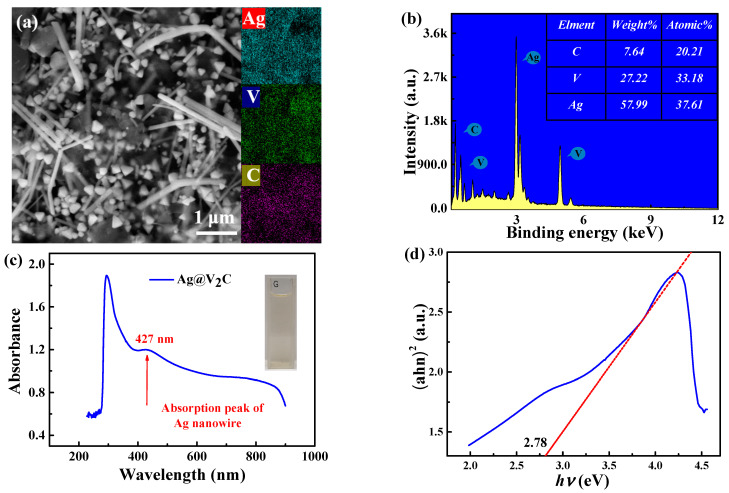
(**a**) SEM and EDS mappings of Ag V C elements. (**b**) EDS of Ag@V2C. The inset is atomic ratios for diverse elements. (**c**) Optical absorption spectra of Ag@V_2_C. (**d**) Estimation of the band gap of Ag@V_2_C.

The Ag@V_2_C hybrids are made ready by mingling the V_2_C hybrid dispersion and AgNO_3_ solution. These are shown in Figure 1. First, 3 mL of original V_2_C colloidal solution (1 mg/mL) in 30mL of AgNO_3_ solution (1 mg/mL) is re-dispersed, and the mixture is sonicated for 30 min. Then, the obtained colloidal solution of hybrid nanocomposites of Ag@V_2_C hybrids is centrifuged at 12,000 rpm for 20 min and re-dispersed in 30 mL deionized water.

### 2.2. Optical Experimental Setup

SEM on a ZEISS Sigma 300 (acceleration voltage: 15 kV, Dublin, CA, USA) was employed to perform the morphological study with a spectrometer (Ocean Optics 4000, CA, USA) to survey the UV–Vis–NIR spectra of monolayer V_2_CT_X_ MXene. To evaluate the NLO features of V_2_CT_X_ MXene flakes nanoparticles, we performed an experiment of a single beam open aperture (OA) Z-scan, with the help of a 6 ns 8 Hz Q-switched Nd:YAG nanosecond pulse laser (Surelite II, Continuum, San Jose, CA, USA) and an optical parametric oscillator (Continuum, APE OPO) to produce lasers beams of diverse wavelengths. In this experiment, the linear transmittance of the V_2_CT_x_ hybrids solution was measured at 500 nm wavelength, and we found that it was 72%. 

The laser beam with a waist diameter of 200 μm is focused through a lens with a focal length of 20 cm and projected onto a 2 mm diameter quartz cuvette filled with aqueous dispersion of V_2_CT_X_ monolayer flake nanoparticles. The cuvette is installed on a computer-operated translation motion stage where the transmitted data for every z point is recorded by stored program. Their NLO properties are studied by Z-scan technology under 532 nm nanosecond laser pulses. The findings indicate that the Ag@ V_2_CT_X_ has a large nonlinear absorption coefficient.

## 3. Results

Figure 1a illustrates the typically morphologic multilayer Ag@V_2_C with coarse surface and the shape of accordion. The elements mapping data of SEM in combination with EDS illustrates that the Ag, V and C elements are evenly dispersed in the monolayer V_2_C. In Figure 1b, the fundamental analysis of Ag@V_2_C V_2_C, the result of C: V: Ag ≈ 2:3:3 is obtained via the energy dispersive X-ray energy spectrum (EDS), while it is impracticable to measure the content of other elements by EDS due to the inadequate content. 

In Figure 1c, the linear absorbance spectra of Ag@V_2_C is investigated by using ultraviolet visible near infrared spectrophotometer to observe two absorption peaks—that is, 315 and 427 nm. These findings justify that the surface of Ag@V_2_C is functionalized with Ag nanoparticles, and the positions of the peaks were predicted by theoretical calculations. In Figure 1d, the estimated band gap value of Ag@V_2_C is 2.75~2.80 eV according to Kubelka-Munk’s theory in previous data [19].

**Figure 2:**
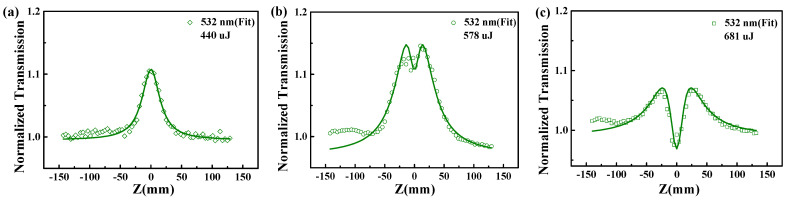
Open aperture Z-scan is conducted at the wavelength of 532 nm and the laser energies at (**a**) 440 μJ, (**b**) 578 μJ and (**c**) 681 μJ to obtain the normalized transmission of V_2_CT_X_ hybrid colloid.

Hereinafter are the three different phases. In Figure 2a, the transmittance of Ag@V_2_C hybrids remains flat before rising sharply when the Z position approaches zero, and a peak is observed, which reflects the SA property. In Figure 2b, the conversion from SA to RSA is observed. The transmittance goes up to the first peak to indicate SA property and then falls back to a valley to indicate the reverse saturable absorbtion RSA property when the Z position approaches zero. It then rises again to the second peak and falls back to the flat linear transmittance. In Figure 2c, the laser pulse energy mounts to 681 when a deep valley occurs, which indicates the RSA property. Figure 2a–c, in general, reveals a whole process of NLO properties, including SA, conversion and RSA. 

**Figure 3:**
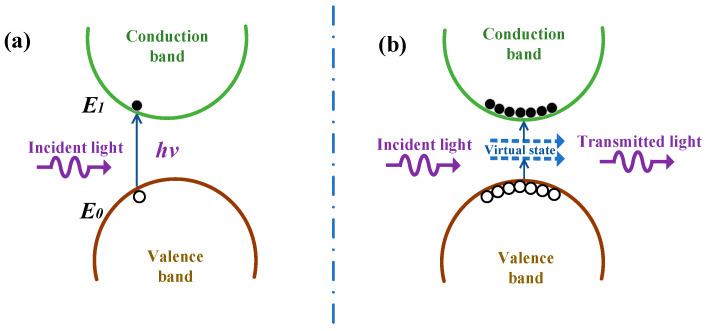
The physical mechanism of the NLO properties of Ag@V_2_C monolayer flakes. (**a**) One photon absorption. (**b**) Two photon absorption.

The physical mechanism of NLO properties can be expounded as follows in Figure 3. The above findings are related in the main to the changing energy of incident laser pulse and the nature property of MXene. It is generally believed that nonlinear susceptibility of the material results from intraband transition, interband transition, hot electron excitation and thermal effects. [20]. Specifically, when Ag@V_2_C monolayer flakes are at low pulse energy, one photon absorption arises to indicate the SA property [21]. 

Under laser irradiance, as the sample approaches the focal point, the laser energy increases abruptly, and the materials absorbs much incident light pulse when Ag@V_2_C monolayer hybrids are pumped into an excited state, leaving a small amount at the ground state. Thus, more light penetrates the sample to make the transmittance higher and SA can be observed [22]. This phenomenon is termed ground-state bleaching of the plasmon band.

To remove the thermal effect’s influence on the possibility of nonlinearity, a laser pulse was set with a short duration and low repetition rate (10 Hz and 6 ns). Moreover, as the incident pulse energy goes up to 1080 μJ, the conversion appears; as it reaches 1380 μJ, RSA occurs. The major causes of RSA may be due to the excited state absorption (ESA) and two-photon absorption (TPA) [23]. 

The quantitative findings of the Z-scan experiment are as follows. The quantitative relationship between laser energy intensity and optical path length can be expressed as follows [24]:

(1) dI=−αIdz

In Equation (1), I signifies the laser intensity, z signifies the optical path length, and α signifies the absorption coefficient. The absorption coefficient combining SA and RSA in Ag@V_2_C monolayer hybrids is expressed in Equation (2) [25]:

(2) $$\alpha (I)=\frac{\alpha_{0}}{1+(I/I_{s})}+\beta I$$

Here, $\alpha_{0}$ signifies the linear absorption coefficient of Ag@V_2_C monolayer hybrids, I signifies the laser intensity, $I_{s}$ is the saturable intensity, and β is the positive nonlinear absorption coefficient. I in Equation (3) can also be expressed as follows in Equation (3):

(3) $$I=\frac{I_{0}}{1+z^{2}/z_{0}^{2}}$$

Therefore, Equation (2) can be reconsidered as:

(4) $$\alpha (I_{0})=\frac{\alpha_{0}}{1+\frac{I_{0}}{(1+z^{2}/z_{0}^{2})I_{s}}}+\frac{\beta I_{0}}{1+z^{2}/z_{0}^{2}}$$

With the help of Equations (1)–(4), the data of normalized transmission in Figure 3 can be fitted. In this way, related parameters are obtained as shown in Table 1.

**Figure 4:**
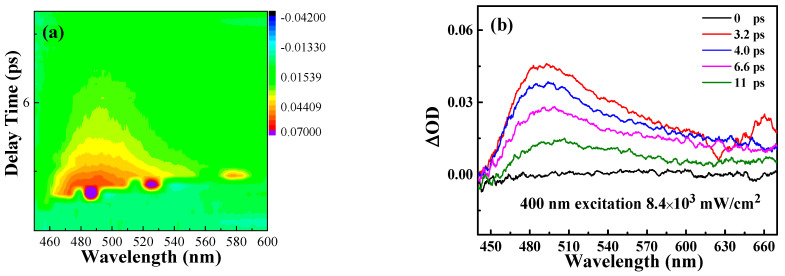
Carrier dynamics (at 400 nm pump) curves for Ag@V_2_C. (**a**) Two-dimensional (2D) mapping of transient absorption spectra pumped at 400 nm with a fluence of 8.4 × 10^3^ mW/cm^2^, (**b**) Time and wavelength resolved transient absorption data of Ag@V_2_C.

To inspect the ultrafast carrier dynamics of Ag@V_2_C hybrids, broadband transient absorption was studied. In Figure 4a, TA spectra of Ag@V_2_C hybrids are illustrated by 2D map of TA signals that have been achieved temporally and spectrally. A constant pump fluence (6.4 × 10^3^ mW/cm^2^) and probe beam ranging from 450 to 600 nm are employed to measure broadband TA signals and obtain the 2D color coded maps, cut horizontally through each map five times to obtain five differential absorption spectra at different delay times (0, 3.2, 4.0, 6.6 and 11 ps). 

In Figure 4b, a positive absorption proves the generation of excited state absorption (ESA) in the spectral region as well as an ultrafast carrier relaxation process within the scale of ps. When the delay time increases, the amplitude of TA spectrum is reduced. The black curve, around the 0 ps delay time, signifies the unexcited Ag@V_2_C hybrids. The peak value of Ag@V_2_C hybrids, 485 nm, forms when the photo-induced absorption is brought about by the conversion of occupied–unoccupied states, which are found in the Z-scan experiment as RSA [26,27,28]. 

Then, the TA signal decays at ~13 ps and falls back to zero in the full waveband. The energy of the pump light markedly exceeds the energy bandgap of the Ag@V_2_C nanosheet, the former 400 nm (~3.10 eV) laser, the latter being ~2.78 eV). Hence, with the incident laser pulse working up, electrons are excited and jump back and forth to the conduction band, while the holes remain in the valence band [29]. 

Soon afterwards is the conversion from electrons to hot electrons through photo-exciting with Fermi–Dirac distribution. The hot carriers will chill down shortly in sync with the decay process through e–e and e–ph diffusion on the conduction band, and with the carrier-phonon dispersing over a few relaxation processes, the hot carriers relax from the conduction band to the valence band and reunite with the holes.

**Figure 5:**
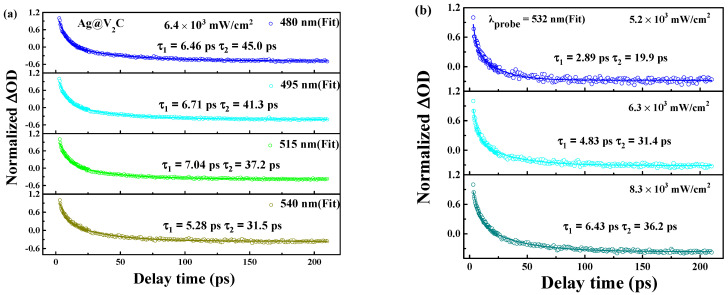
(**a**) Carrier dynamics (at 400 nm pump) curves for Ag@V_2_C nanosheet at various probe wavelengths of 480, 495, 525, 515 and 540 nm, respectively. The scatters are experimental data while the solid lines are theoretical fit generated with pump fluence fixed at 6.4 × 10^3^ mW/cm^2^. (**b**) Carrier dynamics curves (at 400 nm pump) at different pump fluences 5.2 × 10^3^, 6.3 × 10^3^ and 8.3 × 10^3^ mW/cm^2^ with a probe wavelength fixed at 532 nm.

In Figure 5a, the carrier dynamics are inspected at various wavelengths—that is, 480, 495, 515 and 540 nm, as shown in the figure. The optical transmission responses of Ag@V_2_C hybrids are composed of two decay processes, a fast and a slow one. Coulomb-induced hot electrons are excited to be at the core state and be trapped by the surface state before releasing spare energy by dispersing the optical phonons (3.9 ps). The rest of the chilled electrons will experience nonradiative transition to fall back to ground state within 30.1 ps [30,31]. The above-mentioned biexponential decay function is formulated in Equation (3) [32].

(5) $$\frac{\Delta T}{T}=A_{1}\exp(-\frac{t}{\tau_{1}})+A_{2}\exp(-\frac{t}{\tau_{2}})$$

where *A*_1_ and *A*_2_ signify the amplitudes of the fast, slow decay components, respectively; and *τ*_1_ and *τ*_2_ are the decay lifetimes of each component, correspondingly. The experimental data in Figure 5a justify the formulation of Equation (3) through which the fast and slow decay components, *τ*_1_ and *τ*_2,_ are determined along with the rise of probe wavelengths owing to electrons at the lower energy states that are more apt to be detected and whose number is less likely to decrease than those at higher energy states. Similar properties can be seen in graphite [33].

The pump fluence effect on carrier dynamics is inspected at the 500 nm probe wavelength. In Figure 5b, the findings of various pump-fluences (5.1 × 10^3^, 6.4 × 10^3^ and 8.1 × 10^3^ mW/cm^2^) accord with the results by applying Equation (3) and the corresponding parameters. With the rise of pump fluence, *τ*_1_ goes up from 3.9 to 4.5 ps, and *τ*_2_ goes from 11.1 to 13.2 ps, which is inseparable to the carrier density and its reliance on e–ph coupling [34]). 

In brief, the faster decay part of 2D materials follow the principle of e–ph scattering, while the slower part that of ph–ph scattering [35], among which the necessary basis of the electrons’ energy transfer is the electron–phonon interaction [36]. Ultimately, the improved efficiency of electron–phonon interaction promotes the cooling process, or in other words, it is high energy injection that speeds up electron decay. Similar properties can be seen in either quantum dots or 2D films [37,38,39].

The data shown in Figure 5 can be verified upon checking Equation (3) and please see in Table 2 regarding *τ*_1_ and *τ*_2_.

Resulting from the high density of Ag, those aroused electrons in the CB of Ag@V_2_C hybrids tend to move to the d band of Ag atoms until the bleaching effect of VB disappears, which extends the aroused, composite electrons’ lifetime and, accordingly, leads to more violent RSA properties [40]. Next, the electrons in the d band become excited functional groups to take in incident light before they move to a higher energy level and lead to an improved RSA effect. Then, the carriers that are excited and dressed with Ag nanoparticles will move from CB of Ti_3_C to the sp band of the metal until they reach VB of Ag@V_2_C hybrids. Compared with direct decay of pure Ti_3_C_2_ nanosheet, this takes a longer time scale (50 ps), and thus it enhances the RSA performance. Similar properties can be seen in other 2D materials [41].

**Figure 6:**
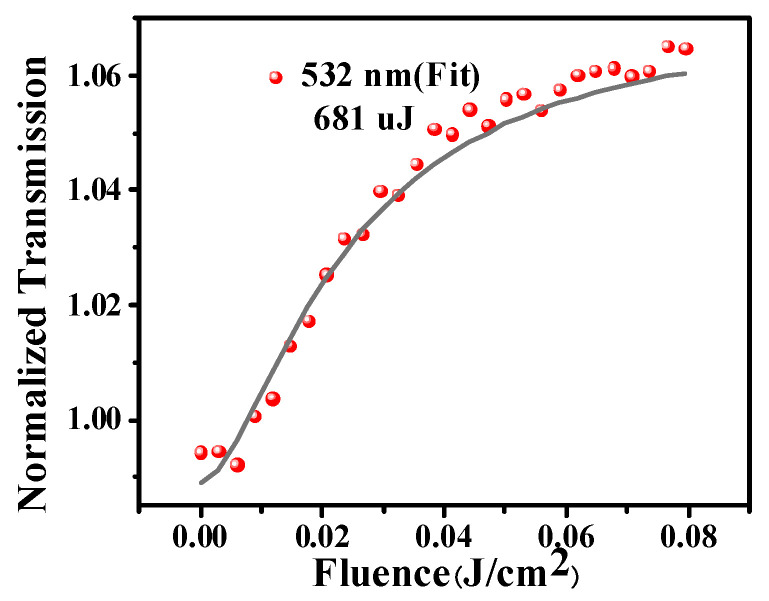
The optical limiting response at 532 nm.

Figure 6 exhibits the optical limiting response at 532 nm. With the increase of incident energy, the transmittance increases rapidly at first. When the incident energy continues to increase, the transmittance does not change drastically, which indicates that Ag@V_2_C has superior optical limiting ability and can be used to manufacture optical limiting devices.

## 4. Conclusions

Ag@V_2_C ultrathin hybrids were synthesized via conventional etching, and remarkable NLO properties of Ag@V_2_C hybrids were found through the OA z-scan technique at 532 nm. SA and RSA properties resulted mainly from GSB and TPA. Moreover, femtosecond transient absorption spectroscopy was adopted to inspect the ultrafast dynamics of the specimen, and we found that its decay contained a fast decay component (~4.5 ps) resulting from electron–phonon interactions and a slow component (~40 ps) from phonon–phonon interactions. Additionally, the two decay times increased with the pump fluence. The experiments confirmed that Ag@V_2_C hybrids can be used in ultrafast optoelectronics and optical limiters.

## Figures and Tables

**Table 1:** The nonlinear optical parameters of Ag@V2C monolayer flake hybrids.

λ (nm)	$I_{0}$ (W/m^2^)	$I_{s}$ (W/m^2^)	β (m/W)
532	1.1 × 10^14^	0.61 × 10^6^	
	1.4 × 10^14^	0.82 × 10^6^	-
	7.4 × 10^13^	0.23 × 10^6^	1.12 × 10^−10^

**Table 2:** Carrier dynamics parameters of the Ag@V_2_C nanosheet.

	λ (nm)	*τ*_1_ (ps)	*τ*_2_ (ps)
Ag@V_2_C	470	4.5	33.9
	485	4.6	36.5
	500	4.2	43.1
	520	3.9	45.8
V_2_C nanosheet	470	3.8	18.0
	485	3.2	27.4
	500	4.6	22.5
	520	4.6	19.9

## Data Availability

Data sharing is not applicable to this article.
